# The Theory of Planned Behaviour in Predicting Determinants of Male Involvement in Reproductive Health Services

**DOI:** 10.24248/eahrj.v9i2.845

**Published:** 2025-12-24

**Authors:** Nyasiro S. Gibore, Fatma Ali Issa, Deogratius Bintabara

**Affiliations:** a Department of Public Health, School of Nursing and Public Health, The University of Dodoma, Tanzania; b Department of Community Medicine, School of Medicine and Dentistry, The University of Dodoma, Tanzania; c Department of Health Promotion, Ministry of Health, Zanzibar, Tanzania

## Abstract

**Background:**

Male involvement in reproductive health services can facilitate timely utilization of these services by women, potentially reducing maternal mortality. This study examined the determinants of male involvement in reproductive health services, guided by the Theory of Planned Behaviour.

**Methods:**

A cross-sectional study was conducted using an interviewer-administered structured questionnaire. Data were collected from 366 randomly selected married men aged 20 years and above. Descriptive statistics and multiple regression analysis were used to examine the strength of the association between the dependent and independent variables.

**Results:**

The odds of male involvement were three times higher for men who showed intention compared to those who did not show intention to be involved in reproductive health services (AOR 3.414, 95% CI, 1.519, 7.674). The odds of male involvement in reproductive health services were 57% lower for men who lived in rural areas when compared with their counterparts (AOR 0.426, 95 % CI, 0.237, 0.768). The odds of male involvement in reproductive health services were 71% lower for men with two wives compared to those with one wife (AOR 0.285, 95 % CI, 0.106, 0.765).

**Conclusion:**

Male involvement in reproductive health services was higher among men who intended to participate, but lower among those living in rural areas and those with two wives. The findings suggest that efforts to improve male involvement in reproductive health services should focus on strengthening men's intention to participate and addressing contextual barriers faced by men in rural and polygamous households. Tailored community-based strategies targeting these groups may enhance uptake and support broader reproductive health outcomes.

## BACKGROUND

Reproductive health services (RHS) encompass access to information and services on prevention, diagnosis, counselling, treatment and care, ensuring safe and timely access for all.^1^ Male involvement in reproductive health (RH) lacks a universal definition but generally refers to men's active participation in antenatal care (ANC), financial support for pregnancy and childbirth, decision-making regarding maternal care, and providing emotional, physical, and financial support to their partners.^2,3^ The 1994 International Conference on Population Development recognised men's influence on women's RH through their role as partners, fathers and healthcare workers with impacts that can be biological, social, direct, or indirect.^4,5^

Globally, men often make key decisions about family size, healthcare access, sexually transmitted infections (STIs) prevention and allocation of household resources.^6,7^ In the Zanzibar community, women traditionally manage productive and reproductive responsibilities, while men control household resources and decisions, including access to RHS.^8^ Male involvement can improve maternal and child health outcomes by supporting birth preparedness, appropriate service utilisation and response to obstetric emergencies. Studies have linked male participation to increased use of antenatal and postnatal services, better nutrition, reduced maternal workload and emotional support.^9–11^

Despite men's decision-making authority, their involvement in RHS in Tanzania remains low.^12,13^ While cultural norms and social roles often discourage male participation,^14^ supportive social networks, especially partners, can improve maternal and child health outcomes.^15–17^ In Zanzibar, RHS has historically focused on women and children, and male involvement is often perceived as a foreign concept. Understanding the factors that influence male involvement is, therefore, critical for designing culturally appropriate interventions.

The determinants of male involvement in RHS vary across contexts, including sociodemographic, cultural, and health system factors.^18–21^ However, little is known about psychosocial determinants, such as intention and social norms, influencing male involvement in Zanzibar. The Theory of Planned Behaviour (TPB) offers a framework to systematically explore these factors.^22^ According to TPB, an individual's intention to perform a behaviour is influenced by their attitude, perceived subjective norms (perceived peer influence) and Perceived Behavioural Control.^22^

In this study, male involvement was assumed to depend on men's intentions, which are influenced by their attitudes toward involvement, their perceptions of peer and community expectations, and their confidence in their ability to participate. Men are more likely to be involved in RHS if these perceptions are favorable.^22^ Identifying these determinants using TPB can inform culturally tailored interventions. Therefore, this study aimed to assess the determinants of male involvement in RHS among men in Zanzibar.

## MATERIAL AND METHODS

### Study Area and Design

This cross-sectional, community-based study using a quantitative approach was conducted on Unguja Island, Zanzibar, Tanzania, from June 2020 to February 2021, covering the South, North, and Urban West districts. Zanzibar is a conservative, Sunni Muslim society with a history shaped by Arabs, Persians, Indians, Portuguese, the British, and the African mainland. This cultural and ethnic diversity provided an ideal context to examine male involvement in reproductive health services (RHS). The study employed the Theory of Planned Behaviour to explore men's intentions, attitudes, subjective norms, and perceived behavioural control regarding RHS.

### Study Population, Inclusion and Exclusion Criteria

The study included married men in Unguja with children aged five years or younger who lived with their partners, were available during data collection, and provided written consent. Focusing on fathers of young children allowed the study to capture recent experiences with RHS. Men who were mentally or severely ill at the time of data collection were excluded.

### Sample Size Calculations and Sampling Procedures

The sample size was estimated using the Leslie Kish formula for cross-sectional studies. Calculations were based on a 95% confidence interval, a 5% margin of error, and an estimated prior prevalence of male involvement in maternity care of 20.3%, as reported in a previous study in Dodoma, Tanzania.^13^ A design effect of 1.5 and an anticipated attrition rate of 5% were also applied, resulting in a final sample size of 392 participants. A multistage sampling procedure was employed. In the first stage, three regions were randomly selected from five using a simple lottery method. In the second stage, one district from each selected region was chosen, resulting in a total of three districts. In the third stage, five Shehias from each district were selected by lottery, giving a total of 15 Shehias. In the fourth stage, systematic random sampling was used to select 26 households from each Shehia, yielding a total of 390 households across the study. The list of eligible households in each Shehia was obtained from the Village Executive Officer's office. Systematic sampling was then applied using a sampling interval of four. The starting household was selected using a random number table, with the starting point chosen by randomly pointing at the table while eyes were closed. Every fourth household thereafter was included until the required sample size was reached. The direction of movement through the Shehia was determined by randomly selecting options written on pieces of paper. Of the eligible participants invited, only 6.2% declined participation, indicating a low refusal rate and minimal risk of non-response bias.

### Data Collection Methods and Tools

Data were collected using an interviewer-administered structured questionnaire adapted from previous study ^23^ and modified for this study based on the TPB and a literature review. The questionnaire consisted of six sections and included both closed- and open-ended questions. The sections covered sociodemographic characteristics, level of male involvement, intentions, perceived attitudes toward male involvement, perceived subjective norms, and perceived behavioural control. The questionnaire was translated into Swahili and pretested in Central District, which has similar characteristics to the study area but was not included in the main study. Necessary revisions were made based on pretest feedback. Four research assistants, each holding a diploma in nursing with over two years of experience in reproductive health services, were trained for three days on the questionnaire and data collection procedures.

### Measurement of Variables

Male involvement in RHS was the dependent variable, assessed through seven specific activities: accompanying a partner to ANC or labour, providing financial support, assisting with domestic work during maternity, and accompanying a partner for child immunisation, growth monitoring, or family planning services. Each activity was scored as ‘1’ (yes) or ‘0’ (no), and the total score was expressed as a percentage. Respondents with a score of 75% or higher, indicating participation in at least five of the seven activities were classified as having high male involvement, while those scoring below 75% were considered to have low male involvement. This threshold is consistent with previous study,^24^ allowing for comparability of findings.

Independent variables included respondents’ sociodemographic characteristics as well as the four constructs of the TPB: intention, perceived attitude, perceived subjective norms, and perceived behavioural control.

### Scoring approach for all TPB constructs

For multi-item scales, responses were dichotomised (Yes = 1, No = 0), and total scores were converted to percentages. Scores ≥50% were classified as high/positive, while scores <50% were classified as low/negative. Attitude items on a four-point Likert scale (1 = strongly disagree to 4 = strongly agree) were dichotomised into “agree” (3–4) and “disagree” (1–2). This approach aligns with previous TPB and Knowledge, Attitude, and Practices (KAP) studies.^25,26^

### Intention to be Involved in Reproductive Health Services

Intention to participate in reproductive health services was assessed using seven statements. These measured whether respondents would accompany their partner to antenatal care and the labour room, provide financial support, assist with domestic work, and participate in the child's immunisations, growth monitoring, and family planning services.

### Perceived Attitude Toward Male Involvement in Reproductive Health Services

Perceived attitude was assessed using seven statements rated on a four-point Likert scale, (1 = strongly disagree, 4 = strongly agree). The statements covered key aspects of male involvement in reproductive health. These included accompanying a partner to ANC services or the labour room, providing financial support for reproductive health services, assisting with domestic work, participating in the child's immunisation and growth monitoring, and engaging in family planning services.

### Perceived Subjective Norms Related to Male Involvement in Reproductive Health Services

Perceived subjective norms regarding male involvement in reproductive health services were assessed using thirteen dichotomous items that measured the extent to which such involvement is considered socially acceptable and supported. Sample items included whether it is acceptable for a man to accompany his partner to ANC or family planning (FP) services, to assist with domestic tasks during pregnancy, and whether family and friends view accompanying a pregnant partner to ANC as normal. Additional items assessed whether a man who participates in reproductive health activities is controlled by his partner or acting out of jealousy.

### Perceived Behavioural Control Related to Male Involvement in Reproductive Health Services

Perceived behavioural control was measured using seven items assessing men's confidence and perceived ease in engaging in reproductive health–related activities. Specifically, participants were asked how easy they found it to: accompany a partner to ANC, delivery, child immunisations, and growth monitoring; attend FP services; assist with domestic chores during pregnancy; and provide financial support for RHS. Responses were used to capture the extent to which men felt capable of participating in these key aspects of reproductive health involvement.

### Data Processing and Analysis

The collected data were entered, coded, and cleaned using SPSS Version 25, then exported to STATA Version 12.0 for analysis. Descriptive statistics, including frequencies, percentages, and medians, were computed and presented in tables and figures. Inferential statistics were used to assess associations between dependent and independent variables.

Multiple logistic regressions were performed to generate odds ratios with 95% confidence intervals, with statistical significance set at *P* < .05. Model fit was evaluated using the Omnibus Test of Model Coefficients, which assesses whether the included predictors improve prediction compared to a model without predictors. The final model included three variables and was statistically significant, χ^2^ (3) = 25.436, *P*= .005, indicating that the predictors collectively improved the classification of male involvement in RHS.

Explanatory power was assessed using Cox & Snell R^2^ (.087) and Nagelkerke R^2^ (.142), suggesting that the predictors explained a meaningful proportion of variance in male involvement, consistent with behavioural and psychosocial research guided by the Theory of Planned Behaviour. The Hosmer–Lemeshow Goodness-of-Fit Test confirmed adequate model fit, χ^2^ (8) = 6.21, P = .623, indicating good agreement between predicted probabilities and observed outcomes.

### Ethical Consideration

The study received ethical approval from the University of Dodoma Institutional Research Review Committee (Ref. No. MA/84/261/02/105) on 31 December 2020. Authorisation to collect data in the districts was granted by the Zanzibar Ministry of Health and Social Welfare (Ref. No. ZAHREC/04/ST/JAN/2021/04). At the Shehia level, permission was obtained from the Village Executive Officer's office. Informed consent either written or verbal was obtained from all participants prior to data collection. Participants were assured of the confidentiality of their responses, and their right to decline or withdraw from the study at any time was clearly communicated.

## RESULTS

### Characteristics of the Respondents

Table 1 presents sociodemographic characteristics of the 366 married men interviewed and included in this analysis. The median age of respondents was 38 years (interquartile range [IQR]: 30–46). Approximately 67% lived in rural areas, while fewer than 8% had attained the highest level of education (college or university). Nearly one-quarter of respondents reported having more than one wife, and about one-third had five or more children.

**TABLE 1:** Percentage distribution of baseline characteristics among men, Ungula, Zanzibar, January 2021 (n = 366)

Variable	n (%)
Age (median = 38, IQR = 30 – 46)	
< 35 years	142 (38.80)
35 – 44	122 (33.33)
45 – 59	68 (18.58)
≥ 60	34 (9.29)
Education level	
No formal education	64 (17.49)a
Primary	127 (34.70)
Secondary	147 (40.16)
Higher	28 (7.65)
Residence	
Urban	122 (33.33)
Rural	244 (66.67)
Occupation status	
Government employed	73 (19.95)
Self-employed	293 (80.05)
Number of wives	
One	276 (75.41)
Two	68 (18.58)
Three	19 (5.19)
Four	3 (0.82)
Number of children (median = 4, IQR = 2 – 6)	
1 – 4	211 (57.65)
5 – 9	114 (31.15)
≥ 10	41 (11.20)

### Male Involvement in Reproductive Health Services

Table 2 shows the percentage distribution of activities used to assess male involvement in RHS. The majority of respondents (70.8%) reported providing financial support to their partners for RHS. Half of the respondents helped their partners with domestic work. More than one-third accompanied their partners for ANC (38.5%), child immunisation (29.2%), and growth monitoring (33.1%). Fewer accompanied their partners for family planning services (22.4%), and only 4% reported accompanying their partners to the labour room.

**TABLE 2:** Percentage distribution of activities assessed male involvement in reproductive health services, Unguja, Zanzibar, January 2021 (n = 366)

Variable	n (%)
Accompany partner to ANC	141 (38.50)
Accompany partner in labour room	14 (3.80)
Provided financial support to the partner for RH	259 (70.80)
Helped his partner domestic chores	185 (50.50)
Accompany partner to child’ immunization	107 (29.20)
Accompany partner for growth monitoring of a child	121 (33.10)
Accompany partner for family planning services	82 (22.40)

### Prevalence of Male Involvement in Reproductive Health Services

**FIGURE 1:**
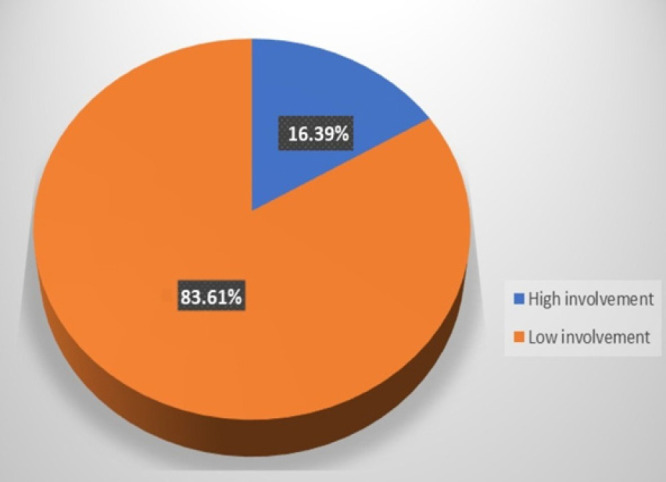
Percentage Distribution of Male Involvement in RH Services, Unguja, Zanzibar, January 2021 (n=366))

Out of 366 respondents, 60 (16.39%) were involved for at least 75% of the seven activities used to measure level of male involvement in RHS. These men were considered to have high male involvement in RHS compared to those who scored less than 75% (Figure 1).

### Respondents’ Intention to Be Involved in Reproductive Health Services

Table 3 presents the percentage distribution of items used to assess male intention to participate in RHS. Most respondents expressed an intention to accompany their partners to ANC (85%), provide financial support for RHS (82%), and attend growth monitoring for their children (74%). However, more than half did not intend to accompany their partners to the labour room. Overall, about 70% of respondents intended to participate in at least 50% of the activities assessed.

**TABLE 3:** Percentage distribution of items assessed male intention to be involved in reproductive health services, Unguja, Zanzibar, January 2021 (n = 366)

Variable	n (%)
Would you accompany your partner to ANC?	310 (84.70)
Would you accompany your partner in labour room?	167 (45.63)
Would you provide financial support to your partner for RH?	301 (82.24)
Would you help your partner with domestic chores	232 (63.39)
Would you accompany your partner during child’ immunization?	242 (66.12)
Would you accompany your partner for growth monitoring of your child?	271 (74.04)
Would you accompany your partner for family planning services?	202 (55.19)
Those who reported “Yes” at least 50% of all the items	259 (70.77)

### Respondents’ Perceived Attitude Toward Involvement in Reproductive Health Services

Table 4 shows the percentage distribution of items used to assess male attitudes toward involvement in RHS. About one-quarter of respondents held a positive attitude toward accompanying their partners to ANC (26.8%) and the labour room (24.3%). Fewer than half expressed positive attitudes toward other activities. Overall, approximately 67% of respondents demonstrated a positive attitude for at least 50% of the items assessing male attitudes toward involvement in RHS.

**TABLE 4:** Percentage distribution of items assessed perceived attitude toward involvement in reproductive health services, Unguja, Zanzibar, January 2021 (n = 366)

Variable	n (%)
A man should accompany his partner to ANC	98 (26.78)
A man should accompany his partner in labour room	89 (24.32)
A man should provide financial support to his partner for RH	119 (32.51)
A man should help his partner with domestic chores	130 (35.52)
A man should accompany his partner during child’ immunization	135 (36.89)
A man should accompany his partner for child's growth monitoring	145 (39.62)
A man should accompany his partner for family planning services	161 (43.99)
Those who reported “Yes” at least 50% of all the items	245 (66.94)

### Respondents’ Perceived Subjective Norms Regarding Male Involvement in Reproductive Health Services

Table 5 presents the distribution of items assessing perceived subjective norms. Approximately 75% of respondents reported that it was acceptable in their community for a man to accompany his partner to ANC. However, about half indicated that their community, family, and friends considered male involvement in RHS unacceptable. More than half agreed that accompanying a partner to immunisation, growth monitoring, and family planning and helping during pregnancy and after delivery was acceptable. About half of respondents perceived that men might be seen as jealous or dominated by their partners if they accompanied them to RHS. Overall, about 60% of respondents reported positive subjective norms for at least 50% of the items.

**TABLE 5:** Percentage distribution of items assessed perceived attitude toward involvement in reproductive health services, Unguja, Zanzibar, January 2021 (n = 366)

Variable	n (%)
Is it acceptable for a man to accompany his partner to ANC?	274 (74.86)
Is it acceptable for a man to accompany his partner in labour room?	155 (42.35)
Is it acceptable for a man to accompany his partner to FP services?	208 (56.83)
Is it acceptable for a man to accompany his partner to immunization?	250 (68.31)
Is it acceptable for a man to accompany his partner for child's growth monitoring?	231 (63.11)
Is it acceptable for a man to help his pregnant partner with domestic chores?	191 (52.19)
Is it acceptable for a man to help his partner domestic work after delivery?	200 (54.64)
Is it considered jealousy when a man accompanies his partner in RH services?	189 (51.64)
Is a man being stigmatized when accompany his partner during RH services?	163 (44.54)
Is it considered to be overpowered by a woman when you accompany your partner in RH services?	180 (49.18)
Do health providers’ attitudes toward man accompanying their partner reduce male involvement?	184 (50.27)
Should a man be involved in RH care services?	199 (54.37)
Do your family friends consider it acceptable for you to accompany your pregnant partner in RH services?	177 (48.36)
Those who reported “Yes” at least 50% of all the items	214 (58.47)

### Respondents’ Perceived Behavioural Control Regarding Involvement in Reproductive Health Services

Table 6 shows the distribution of items assessing perceived behavioural control. Nearly half of respondents reported positive perceived behavioural control across all items. Overall, less than half (48.6%) had positive perceived behavioural control for at least 50% of the items.

**TABLE 6:** Percentage distribution of items assessed perceived behaviour control regarding male involvement in reproductive health services, Unguja, Zanzibar, January 2021 (n = 366)

Variable	n (%)
Is it easy to accompany your partner to ANC services?	191 (52.19)
Is it easy to accompany your partner during delivery?	197 (53.83)
Is it easy to accompany your partner for your child's immunization?	179 (48.91)
Is it easy to accompany your partner for your child's growth monitoring?	184 (50.27)
Is it easy to accompany your partner to FP services?	183 (50.00)
Is it easy to help your partner with domestic chores during pregnancy?	193 (52.73)
Is it easy for you to provide financial support for RH services?	212 (57.92)
Those who reported “Yes” at least 50% of all the items	178 (48.63)

### Factors Associated with Male Involvement in Reproductive Health Services

Table 7 presents unadjusted and adjusted logistic regression analyses for factors associated with male involvement in RHS. In the unadjusted model, the number of wives, place of residence, and intention were significantly associated with male involvement. These associations remained significant in the adjusted model. Based on the final logistic regression model, the predicted prevalence of male involvement in RHS was estimated at 22.4%, reflecting the probability of involvement after accounting for significant predictors (intention, residence, and number of spouses). Specifically, the odds of male involvement in RHS were 57% lower for men living in rural areas compared with urban residents (AOR 0.426; 95% CI, 0.237 to 0.768). Married men with two wives had 71% lower odds of involvement compared with those with one wife (AOR 0.285; 95% CI, 0.106 to 0.765). Conversely, men who expressed an intention to be involved were over three times more likely to participate in RHS compared with those who did not (AOR 3.414; 95% CI, 1.519 to 7.674).

**TABLE 7:** Logistic regression analysis for predicting factors associated with male involvement in reproductive health services, Unguja, Zanzibar, January 2021 (n = 366)

Variable	Unadjusted model		Adjusted model	
	OR (95% CI)	P	AOR (95% CI)	P
Respondent's age (as continuous)	1.001 (0.979 – 1.024)	.908		
Number of wives (ref: one)				
Two	0.359 (0.137 – 0.938)	.036	0.285 (0.106 – 0.765)	.013
Three	1.205 (0.384 – 3.786)	.749	1.302 (0.378 – 4.481)	.676
Four	2.260 (0.201 – 25.414)	.509	3.471 (0.250 – 48.174	.354
Residence (ref: urban)				
Rural	0.430 (0.245 – 0.754)	.003	0.426 (0.237 – 0.768)	.005
Education level (ref: none)				
Primary	0.471 (0.216 – 1.028)	.059	0.450 (0.198 – 1.023)	.057
Secondary	0.669 (0.326 – 1.376)	.275	0.590 (0.275 – 1.265)	.175
Tertiary	0.544 (0.163 – 1.819)	.323	0.426 (0.118 – 1.531)	.191
Occupation (ref: employed)				
Self-employed	1.132 (0.556 – 2.304)	.733		
Number of children (as continuous)	0.984 (0.903 – 1.073)	.723		
Intention (ref: No)				
Yes	3.108 (1.422 – 6.795)	.004	3.414 (1.519 – 7.674)	.003
Perceived attitude (ref: No)				
Yes	0.985 (0.548 – 1.773)	.961		
Perceived subjective norms (ref: No)				
Yes	0.844 (0.483 – 1.474)	.551		
Perceived behavioural (ref: No)				
Yes	1.257 (0.778 – 2.366)	.282		

OR=Odds Ratio; AOR=Adjusted Odds Ratio

## DISCUSSION

This study applied the Theory of Planned Behaviour (TPB) to examine determinants of male involvement in RHS among married men in Unguja, Zanzibar, Tanzania. The findings indicate that intention was the only TPB construct that significantly predicted male involvement. This is consistent with TPB's core assertion that intention is the most proximal determinant of behaviour.^27^ Although attitude, subjective norms, and perceived behavioural control were not statistically significant predictors in the regression model, descriptive findings showed that many men reported positive attitudes and supportive subjective norms toward involvement.

These descriptive patterns may help explain the relatively high levels of intention observed, even though the constructs did not independently predict behaviour. The non-significance of these predictors suggests that intention may be influenced more by personal motivations or contextual experiences, such as prior exposure to birth complications or a perceived sense of responsibility, than by perceived social expectations or self-efficacy. A similar study conducted in Tanzania also reported that none of the TPB constructs (attitude, subjective norms, or perceived control) significantly predicted intention to deliver in a health facility, despite generally high intention levels.^26^

These similarities reinforce the idea that, in this context, intention is a crucial lever for behaviour change, even if the underlying TPB constructs (attitudes, norms, control) do not independently predict action in multivariable models. Furthermore, the descriptive prevalence of positive attitudes and subjective norms in our study (e.g., more than half of participants held favourable views) echoes findings from other Tanzanian research in which a baseline assessment in rural Rukwa documented generally positive attitudes and subjective norms among pregnant women toward male involvement.^28^

Taken together, these studies suggest that while social and cognitive predispositions (attitudes and norms) are widely present, intention may serve as the most direct mechanism linking these predispositions to actual involvement. Therefore, interventions designed to increase male participation in RHS should prioritise strengthening men's intentions, such as by reinforcing motivational factors, while also maintaining supportive social norms and enhancing men's control over opportunities for involvement. Given that intention was the sole significant determinant, programmes aiming to increase male involvement should focus on reinforcing men's motivation and commitment to participate in RHS while ensuring supportive environments that make such involvement feasible.

The finding that perceived behavioural control was not associated with male involvement was not surprising, as fewer than half (48.63%) of participants displayed positive perceived behavioural control for at least 50% of the activities assessed. This suggests that most men lacked confidence in their ability to engage in RHS, possibly reflecting past experiences in which men were excluded from reproductive health matters. Historically, such exclusion limited men's capacity to make informed decisions in obstetric emergencies and reduced their interest in participating in RHS.^29^ According to the TPB, the greater an individual's perceived behavioural control, the stronger their ability to perform the behaviour, and this construct depends on the accuracy with which perceived control reflects actual control.^22^ Therefore, additional efforts may be required to strengthen men's confidence in their ability to participate. For instance, making RHS more male-friendly by incorporating services that address men's needs and by providing education on the benefits of male involvement may help increase their perceived control.

This study also found no significant relationship between subjective norms and male involvement in RHS in the multivariate analysis. This is consistent with previous TPB reviews, which have noted that subjective norms often fail to reach statistical significance in predicting health-related behaviours.^30^ Nevertheless, descriptive data showed that more than half of respondents reported positive subjective norms (Table 5). This highlights an important distinction: although subjective norms were generally favourable, they were not strong enough to independently predict behaviour once other variables were considered. The broader social and cultural context may help explain this inconsistency. In Unguja, traditional cultural expectations, strongly influenced by Islamic norms emphasizing modesty, gender separation, and avoidance of mixed-gender spaces, shape everyday interactions. These norms create subtle but persistent pressures that discourage men from participating in RH activities. For instance, a man accompanying his partner to spaces perceived as “women-only”, such as ANC or FP clinics, may be seen as violating community expectations. These pressures, even if not fully captured in quantitative measures, may still indirectly shape behaviour. Similar findings have been reported in other East African studies, which suggest that subjective norms influence RH behaviours through social expectations, gender norms, and normative beliefs, even when statistical associations are weak.^13,31,32^

Regarding demographic factors, the study found that urban residence was associated with higher male involvement in RHS. This aligns with studies from Ethiopia, which reported a link between urban residence and men's involvement in birth preparedness and complication readiness,^33^ and from Ghana, where urban men were more likely than rural men to have children delivered in a health facility.^34^ Urban men may have greater access to information about the benefits of involvement through exposure to RHS services, media, and other information channels that are more readily available in urban settings. Another possible explanation is that urban men are more exposed to modern lifestyles and less bound by cultural traditions discouraging male participation in maternal health. For example, Zanzibari cultural norms traditionally prohibit men from being present during childbirth, based on beliefs that doing so weakens the man. Similarly, men accompanying their wives to antenatal care may be stigmatised as weak or foolish. Such beliefs discourage male participation. Similar findings have been reported in South Africa^35^ and Kenya.^36^ Addressing these cultural barriers is crucial, particularly in rural areas. Outreach interventions targeting both men and women are strongly recommended in areas with limited healthcare access and transportation challenges.

The study also found that men in monogamous marriages were more likely to be highly involved in RHS than men in polygamous unions. This is consistent with findings from other African countries, where monogamous men were significantly more involved in maternal health care compared to their polygamous counterparts.^37^ Likewise, men in monogamous marriages were found to accompany their partners for maternity care at higher rates.^31^ A study in Yangon, Myanmar, similarly reported a negative association between polygamous marriage and male involvement.^38^ Polygamous women may be less inclined to discuss or involve their husbands in maternal health matters, and husbands may struggle to prioritise between multiple partners who may require support simultaneously.^39^

A notable strength of this study is its grounding in a psychological theory to explain men's reproductive health-seeking behaviour. By using this theoretical framework, the study provides deeper insight into the factors influencing male involvement in reproductive health, moving beyond surface-level observations to understand underlying motivations and attitudes. The study also drew participants from multiple age groups and stratified them by urban and rural residence. This approach ensured a broad range of perspectives, reflecting both geographic and generational diversity, which strengthens the richness and applicability of the findings.

Despite these strengths, the study has some limitations. The selection criteria may have encouraged participants to provide socially desirable responses. Additionally, the study excluded single and unmarried men, based on the assumption that they have limited experience with healthcare services and childbirth-related complications. This exclusion may have overlooked important perspectives and experiences, thereby limiting the generalisability of the findings to all men. As a result, while the study offers valuable insights, its conclusions should be interpreted with caution when considering broader populations.

## CONCLUSION

This study demonstrates that the Theory of Planned Behaviour is a useful framework for understanding male involvement in reproductive health services in Unguja. Intention emerged as the only significant psychosocial predictor of involvement. In addition, rural residence and polygamous marriage were identified as key sociodemographic barriers to engagement in RHS.

Although subjective norms did not independently predict involvement, the cultural context suggests that gendered expectations and community norms remain important barriers, particularly in rural areas. Addressing these cultural pressures, while strengthening men's intentions through targeted health education and male-friendly programmes will be essential for improving male participation in RHS. Overall, interventions should focus on increasing men's intentions to participate, reducing geographical and social barriers, and addressing harmful normative beliefs that continue to shape reproductive health behaviours.
